# Development of the roadmap for reducing cardiovascular morbidity and mortality through the detection, treatment and control of hypertension in Africa: report of a working group of the PAS CAR Hypertension Task Force

**Published:** 2016

**Authors:** Anastase Dzudie, Abdoul Kane, Euloge Kramoh, Jean-Baptiste Anzouan-Kacou, Jean Marie Damourou, Lucien Allawaye, Jolis Nzisabira, Latif Mousse, Dadier Balde, Ouane Nouhom, Jean Louis Nkoa,, Kimbally Kaki, Armel Djomou, Alain Menanga, Samuel Kingue, Christ Nadege Nganou, Liliane Mfeukeu Kuate, Jean Bruno Mipinda, Lucie Nebie, Serigne Abdou Ba

**Affiliations:** Douala General Hospital and Buea Faculty of Health Sciences, Douala, Cameroon; Soweto Research Group and National Institute of Health Millennium Fogarty Chronic Disease Leadership programme, Department of Medicine, University of the Witwatersrand, Johannesburg, South Africa; Service de cardiologie, Hôpital Général de Grand Yolf, Dakar, Senegal; Institut cardiologique d’Abidjan, Cote d’Ivoire; Institut cardiologique d’Abidjan, Cote d’Ivoire; Service de cardiologie, Centre Hospitalier et Universitaire de Lomé, Togo; Hôpital Général de Djamena, Chad; Hôpital militaire de Bujumbura, Burundi; Centre Hospitalier de Cotonou, Benin; Centre Hospitalier et Universitaire, Conakry, Guinee Conakry; Centre Hospitalier et Universitaire de Bamako, Mali; Centre Hospitalier et Universitaire, Brazaville, Congo; Centre Hospitalier et Universitaire, Brazaville, Congo; Hôpital Laquintinie de Douala, Cameroon; Cardiology Unit, Department of Internal Medicine, Yaoundé Central Hospital, Yaoundé, Cameroon; Cardiology Unit, Department of Internal Medicine, Yaoundé Central Hospital, Yaoundé, Cameroon; Service de cardiologie, Hôpital Central de Yaoundé, Yaoundé, Cameroon; Service de cardiologie, Hôpital Central de Yaoundé, Yaoundé, Cameroon; Hôpital Universitaire du Centre de Libreville, Gabon; Polyclinique internationale de Ouagadougou, Burkina Faso; Service de cardiologie, Université Cheikh Anta DIOP, Dakar, Senegal

**Keywords:** hypertension, policy, PASCAR, roadmap, Africa

## Abstract

The fourth Pan-African Society of Cardiology (PASCAR) hypertension taskforce meeting was held at the Yaoundé Hilton Hotel on 16 March 2016. Its main goals were to update and facilitate understanding of the PASCAR roadmap for the control of hypertension on the continent, to refine the PASCAR hypertension algorithm, and to discuss the next steps of the PASCAR hypertension policy, including how the PASCAR initiative can be customised at country level. The formation of the PASCAR coalition against hypertension, the writing group and the current status of the PASCAR hypertension policy document as well as the algorithm were presented to delegates representing 12 French-speaking countries. The urgency to finalise the continental policy was recognised and consensus was achieved by discussion on the main points and strategy. Relevant scientific issues were discussed and comments were received on all points, including how the algorithm could be simplified and made more accessible for implementation at primary healthcare centres.

## Abstract

In sub-Saharan Africa, the high prevalence of hypertension is coupled with poor rates of detection, treatment and control.^1^ The disease is a major threat to achievement of the World Health Organisation Global Action Plan 2013–2020 for non-communicable disease (NCD) reduction, specifically focused on heart attacks, strokes and other cardiovascular diseases.

As the leading continental organisation, the Pan-African Society of Cardiology (PASCAR), including all hypertension experts and stakeholders, embarked on the development of a continental hypertension policy to help reduce heart disease and stroke on the continent by the year 2025. To achieve its goal, this coalition started working in 2014,^2^ achieving consensus on a majority of points by group discussion during conference calls and three face-to-face meetings, as well as by iterative revisions of the written document. This document, which is a customisation of the World Heart Federation (WHF) global roadmap^3^ to the Africa-specific context, was discussed and reviewed on several occasions in opportunistic and scheduled meetings.

The Cameroon Cardiac Society (CCS) meeting held in Yaoundé from 16 to 18 March 2016 was a unique occasion to review the progress so far and discuss the next steps with national cardiac societies from French-speaking African countries, some of which were unable to fully participate physically in the previous steps. This opportune meeting welcomed together a large number of delegates representing 12 French-speaking African countries.

## Welcome address

Prof Abdou Serigne BA (Senegal), vice president of the PASCAR West region and chair of the meeting, welcomed the participants and thanked them for their time and the effort made to attend the meeting, and for their enthusiasm in contributing to this unique opportunity to develop a clear policy against hypertension in Africa. He expressed gratitude to the CCS for convening this meeting, which would serve as a call to all other African national cardiac societies to join the continental organisation. He also set out the agenda, presented an update on the PASCAR hypertension roadmap, and made a presentation on the PASCAR hypertension algorithm, which was followed by friendly, open discussion.

As president of the CCS and co-chair of the meeting, Prof Samuel Kingue (Cameroon) followed Prof Ba, welcoming the delegates and wishing the group a fruitful working session. The second co-chair, Prof Jean Louis Nkoua (Congo) joined his peers in welcoming participants and wished them a successful meeting.

## Update on the PASCAR roadmap for hypertension

Dr Anastase Dzudie, chair of the PASCAR Task Force on Hypertension, presented an update on the PASCAR hypertension roadmap thus far and an evidence-based review on clinical trials and guidelines for hypertension management in Africa. The work to date has consisted of three face-to-face meetings (Nairobi: 27 October 2014, London: 30 August 2015, and Mauritius: 3 October 2015) and several conference calls, during which the situation analysis was done, the WHF policy document was presented and discussed, and the continental policy was drafted, taking into consideration the specific African context.

During these working sessions, relevant scientific issues were discussed, comments were received on all points, and consensus was achieved by discussion. The following key stakeholders have collaborated with PASCAR in the developmental process: International Forum for Hypertension Control in Africa, African Heart Network, International Society of Hypertension (low- and middle-income countries), and several national cardiac societies.

## PASCAR algorithm for the diagnosis and management of hypertension

Prof Abdoul Kane (Senegal) presented the PASCAR algorithm for the diagnosis and management of hypertension in the African population. Prof Kane concluded his talk by expressing the willingness of the writing group to receive feedback in order to improve the algorithm.

## Discussion

The delegates were from 12 African countries, including Cameroun, Togo, Cote D’Ivoire, Mali, Gabon, Chad, Senegal, Burkina Faso, Togo, Congo, Benin and Burundi. The audience acknowledged that the lack of evidence on drug trials on the continent, as well as the small number of active hypertension policy programmes on the continent were a real concern needing an urgent response.

The PASCAR response was therefore recognised as a timely and most appropriate one to the challenge of management and prevention of hypertension in the region. The algorithm was welcomed and discussed, and the audience was advised to make it simple and widely available, especially at primary healthcare centres where a substantial proportion of hypertensive patients are managed. Comments on the algorithm were received and feedback will be sent by e-mail.

## Conclusion

The delegates acknowledged the importance of advocacy to continental organisations such as the African Union. Experts recognised that strong government leadership and policy are mandatory to making hypertension a priority in the region, and adopting and implementing a minimum standard for the health system to achieve hypertension control.

**Delegates at the conference:**
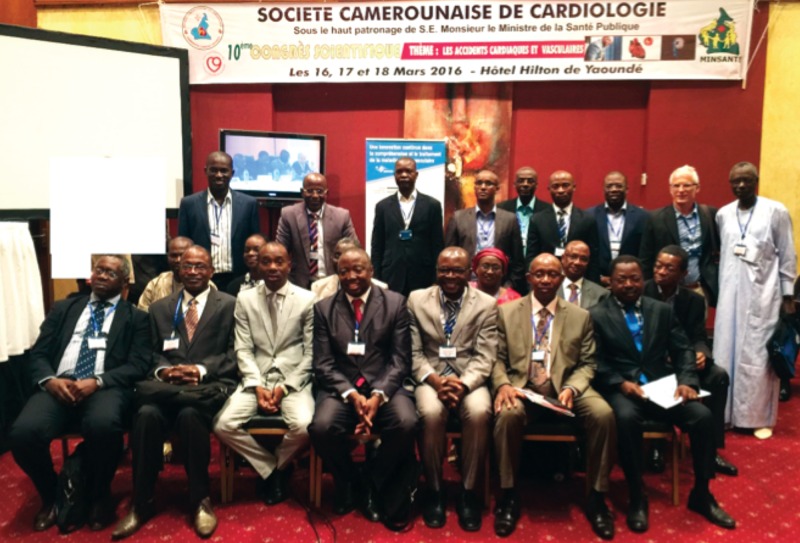
Front (left to right): Ouane Nouhom (Mali), Jean Louis Nkoua (Congo), Abdoul Kane (Senegal), Seringne Abdou Ba (PASCAR council, Senegal), Anastase Dzudie (chair, PASCAR task force on hypertension), Samuel Kingue (president, Cameroon Cardiac Society), Findibe Damourou (president, Togolese Society of Cardiology). Middle (left to right): Adama Kane (Senegal), Edwige Siransy (Côte d’Ivoire), Goeh Akue Edem (Togo), Mariam Béavogui (Guinée), Dadhi Balde (Guinée), Yves Moukam (Cameroon). Back (left to right): Roland N’guetta (Côte d’Ivoire), Aime Bony (Cameroon), Lucien Allawaye (Tchad), Jolis Nzisabira (Burundi), Latif Mousse (Benin), Jerome Boomhbi (Cameroon), Jean-Baptiste Anzouan-Kacou (Côte d’Ivoire), Xavier Jouven (France), Moustapha Sarr (Senegal).
